# Hypothermia Induced Impairment of Platelets: Assessment With Multiplate vs. ROTEM—An *In Vitro* Study

**DOI:** 10.3389/fphys.2022.852182

**Published:** 2022-03-29

**Authors:** Bernd Wallner, Bettina Schenk, Peter Paal, Markus Falk, Giacomo Strapazzon, Wenjun Z. Martini, Hermann Brugger, Dietmar Fries

**Affiliations:** ^1^ Department of Anaesthesiology and General Intensive Care Medicine, Medical University of Innsbruck, Innsbruck, Austria; ^2^ Department of General and Surgical Intensive Care Medicine, Medical University of Innsbruck, Innsbruck, Austria; ^3^ Institute of Mountain Emergency Medicine, Eurac Research, Bolzano, Italy; ^4^ Alexion Pharma Austria GmbH, Wien, Austria; ^5^ Department of Anaesthesiology and Intensive Care Medicine, St. John of God Hospital, Paracelsus Medical University, Salzburg, Austria; ^6^ US Army Institute of Surgical Research, San Antonio, TX, United States

**Keywords:** coagulation, hemorrhage, hypothermia, multiplate, platelets, rotational thromboelastometry (ROTEM)

## Abstract

**Introduction:** This experimental *in vitro* study aimed to identify and characterize hypothermia-associated coagulopathy and to compare changes in mild to severe hypothermia with the quantitative measurement of rotational thromboelastometry (ROTEM) and multiple-electrode aggregometry (MULTIPLATE).

**Methods:** Whole blood samples from 18 healthy volunteers were analyzed at the target temperatures of 37, 32, 24, 18, and 13.7°C with ROTEM (ExTEM, InTEM and FibTEM) and MULTIPLATE using the arachidonic acid 0.5 mM (ASPI), thrombin receptor-activating peptide-6 32 µM (TRAP) and adenosine diphosphate 6.4 µM (ADP) tests at the corresponding incubating temperatures for coagulation assessment.

**Results:** Compared to baseline (37°C) values ROTEM measurements of clotting time (CT) was prolonged by 98% (at 18°C), clot formation time (CFT) was prolonged by 205% and the alpha angle dropped to 76% at 13.7°C (*p* < 0.001). At 24.0°C CT was prolonged by 56% and CFT by 53%. Maximum clot firmness was only slightly reduced by ≤2% at 13.7°C. Platelet function measured by MULTIPLATE was reduced with decreasing temperature (*p* < 0.001): AUC at 13.7°C −96% (ADP), −92% (ASPI) and −91% (TRAP).

**Conclusion:** Hypothermia impairs coagulation by prolonging coagulation clotting time and by decreasing the velocity of clot formation in ROTEM measurements. MULTIPLATE testing confirms a linear decrease in platelet function with decreasing temperatures, but ROTEM fails to adequately detect hypothermia induced impairment of platelets.

## Introduction

Multiple trauma is associated with accidental hypothermia (<35°C) in 12–66% of all cases (Hildebrand et al., 2009; Mommsen et al., 2013). In numerous studies, the injury severity score (ISS) was higher in hypothermic compared to normothermic patients (Martin et al., 2005; Shafi et al., 2005; Bukur et al., 2012; Mommsen et al., 2013; Winkelmann et al., 2019). For instance, in one study the ISS was 35.0 in the patients diagnosed with hypothermia at hospital admission, while it was 29.2 in normothermic patients (Trentzsch et al., 2012). Furthermore, a lower body temperature is independently associated with increased odds of death (odds ratio, 3.03; 95% CI, 2.62–3.51) (Wang et al., 2005).

Hypothermia impairs clotting factor activity and platelet function. In multiple trauma patients these undesirable effects on the coagulation system are intensified by hemorrhage, inflammation, acidosis, but also by therapeutic interventions like volume resuscitation and the administration of drugs, which act directly or indirectly on the coagulation system (Moore et al., 2021). Besides detrimental effects on the coagulation system, hypothermia increases morbidity and mortality in trauma patients (Jurkovich et al., 1987; Wolberg et al., 2004; Gerecht, 2014; Vardon et al., 2016; Ise et al., 2020; van Veelen and Brodmann Maeder, 2021). In mild hypothermia (35°–32°C), coagulopathy results primarily from inhibited platelet adhesion. Wolberg et al. showed that platelet aggregation and adhesion were significantly reduced at 33.0°C compared with 37.0°C (*p* < 0.05) and below 33.0°C, enzyme activity and platelet function were significantly reduced (Wolberg et al., 2004).

The diagnosis of hypothermia-associated coagulopathy is still challenging. The negative effects of hypothermia on plasma coagulation and cellular hemostasis cannot be identified with standard laboratory techniques such as platelet count, prothrombin time (PT) and activated partial thromboplastin time (aPTT), because these parameters are usually measured at the default of 37°C (Martini et al., 2008). Rotational thromboelastometry (ROTEM) is increasingly used as point-of-care analysis of trauma-associated coagulopathy and can better identify trauma associated coagulopathy, already at the bed-side (Johansson et al., 2009; Park et al., 2009; Schöchl et al., 2010; Ise et al., 2020). The ROTEM gamma and delta devices further provide the possibility to measure blood samples at variable temperatures according to the present temperature of the patient. In addition, multiple-electrode aggregometry (MULTIPLATE) has been used recently to assess platelet function and is more frequently being used to assess hypothermia associated impairment in platelets (Kander et al., 2014; Würtz et al., 2014). However, direct comparisons of coagulation alterations with different blood temperatures by ROTEM and MULTIPLATE are not available. In a previous study our research group has evaluated the overall impact of hypothermia on coagulation and have introduced Real Time Live Confocal Microscopy as a new tool to visualize hypothermia-induced impairment of thrombin (Wallner et al., 2020). This *in vitro* follow-up study was designed to focus on the clinical aspects of hypothermia-associated coagulopathy and on the important role of platelets which is evaluated by comparing measurements of ROTEM and MULTIPLATE with low blood temperatures that clinically conform to mild to severe hypothermia.

## Materials and Methods

### Ethics Approval

This study was approved by the institutional review board of the Innsbruck Medical University, Austria (Ref. No. AN2016-0102 362/4.13). Written informed consent was obtained from all study participants. The study was performed in compliance with the Declaration of Helsinki and followed the Good Clinical Practice guidelines as defined by the International Conference on Harmonization (ICH-GCP).

### Population

Healthy, randomly selected volunteers (*n* = 18, nine females, 22–46 years) were enrolled at the Department of Anaesthesiology and General Intensive Care, Medical University of Innsbruck. The population was the same as in the preceding study, however we conducted new and differing calculations performed under a new, clinically oriented focus. Exclusion criteria were pregnancy, breastfeeding, active participation in another clinical trial, any known coagulopathies of the participant, or intake of any kind of medication, including NSAID in the last 14 days.

### Collection and Preparation of Blood Samples

Blood samples were collected and processed as described previously (Wallner et al., 2020). In brief, blood samples were drawn into vacutainer tubes containing 1/10 volume of 3.2% trisodium citrate (Sarstedt, Nümbrecht, Germany) for ROTEM and fibrinogen measurements. Hirudin tubes (Sarstedt, Nümbrecht, Germany) were used for platelet function testing with a Multiplate® analyzer (Roche Diagnostics, Mannheim, Germany). A blood sample was drawn for each temperature and immediately incubated and cooled in five different water baths to the defined temperature. Target temperatures were selected according to cut-off values for accidental hypothermia stages (Swiss staging system) and the lowest ever accidentally acquired and survived temperature at the time of the trial (37, 32, 24, 18, and 13.7°C) (Gilbert et al., 2000; Paal et al., 2016). The temperature of the water-bath was controlled within a deviation of +/−0.1°C. Besides the blood samples, we also cooled the entire laboratory by air-conditioning to prevent a warming of the blood samples during handling and during the experiment. At the ROTEM Gamma machine the maximum temperature can be defined and heating of the analyzer-block can be stopped at this temperature. The various blood samples were incubated for 30 min before being analyzed to reach the target temperatures precisely. The analyses were performed starting with the lowest temperature and ending with the highest temperature to minimize the confounder of platelet storage. The two analyzers underwent all calibrations, checks and periodical quality control tests as required by the producer.

The Swiss staging system was first published in 2003, in a recommendation of the International Commission for Mountain Emergency Medicine (ICAR MedCom) for medical on-site decision making in patients with accidental hypothermia (Durrer et al., 2003). Each stage defined an estimated core temperature range according to clinical findings, based on level of consciousness, breathing and circulation, presence or absence of shivering, and finally apparent death. The Swiss system is a useful tool when a reliable core temperature measurement is not available. A recent article further simplified the field staging when temperature measurement is not available, and included the alert, voice, pain, and unresponsive (AVPU) scale to assess the level of consciousness and an estimate of the risk of cardiac arrest caused by hypothermia (Musi et al., 2021).

### Thromboelastometry

The ROTEM (ROTEM®, Innovations GmbH, Munich, Germany) device temperature was set to the target temperature of the blood samples. After blood draw and incubation for a minimum of 30 min the samples were analyzed with the respective reagents provided by the manufacturer for activation of the extrinsic (ExTEM) and intrinsic (InTEM) pathways of the coagulation cascade and without the effect of platelets (FibTEM). ExTEM is activated by means of a small amount of tissue thromboplastin (Tissue Factor) and is used as a screening test for the (extrinsic) hemostasis system. The effect is influenced by platelets, fibrinogen and extrinsic coagulation factors. InTEM is activated *via* the contact phase. This is therefore sensitive to factor deficiencies in the intrinsic system and to the presence of heparin in the sample. InTEM mildly activates the contact phase of hemostasis. FibTEM test is an ExTEM based test for the evaluation of the fibrin part of the clot. The addition of cytochalasin D blocks the platelets. The resulting clot is thus dependent only on fibrin formation and polymerization.

Standard parameters included coagulation time (CT), i.e., the time until clot formation starts; clot formation time (CFT), i.e., the time from CT until the clot reaches an amplitude of 20 mm; A5 value (A5), indicating the clot amplitude 5 minutes after CT, which is a fast tool for detecting changes in coagulation; maximum clot firmness (MCF), defined as the maximum amplitude reached by clot formation during measurement; and alpha angle (Alpha), which is the angle between the middle axis and the tangent to the clotting curve through the 20 mm amplitude point. The median value per measurement run was used for analysis.

### Platelet Function Testing

Hirudin whole blood samples were incubated for a minimum of 30 min at the respective target temperature. Afterwards, blood samples were analyzed using a Multiplate® Analyzer (Roche Diagnostics, Basel, Switzerland) and initiated using arachidonic acid 0.5 mM (ASPI test), thrombin receptor-activating peptide-6 32 µM (TRAP test) or adenosine diphosphate 6.4 µM (ADP test) according to manufacturer’s instructions. The blood samples were analyzed at the set low temperatures. Given the small quantity of liquid, approx. 600 μL, we cannot rule out any rewarming during the analysis. All tests (ROTEM and Multiplate) were performed in an air-conditioned room to keep the ambient temperature low. For normal operation, the Multiplate analyzer uses a heating bar to warm the blood samples. We bypassed this warming-up phase and started the tests immediately in the cold analyzer. Additionally, we did not add 300 μL of the pre-warmed NaCl 0.9% but a NaCl 0.9%, which had the same temperature as the blood sample to be tested. Test results were expressed as the area under the curve (AUC). Device temperature was set to the respective experimental temperature range (13.7°C–37°C, see above).

### Statistics

Samples size estimation for this trial was based on experimental data described in Ruzicka J. et al. (Ruzicka et al., 2012). We calculated that 18 participants would provide a power of 80% at a 5% significance level to determine the prespecified effect size of 80%, assuming a 10% dropout rate and 10% safety margin. Data is presented as mean and standard deviation and differences to baseline at 37°C are expressed as percentages. Group differences in temperatures were assessed by means of an analysis of repeated measures (ANOVA) using a mixed model with temperature as repeated effect assuming an unstructured covariance matrix, followed by Bonferroni corrected post-hoc tests. Normality at 37°C was assessed visually and by means of Shapiro-Wilk-test. *p* values are two-sided and values <0.05 were considered statistically significant and IBM SPSS Statistics for Windows, Version 21.0. Armonk, NY: IBM Corp. was used for analysis.

## Results

### Population Characteristics

We recruited the same 18 volunteers that have already participated in the preceding study, nine were female and nine male. Mean age was 31 years (range 22–46 years), height 174 cm (158–186 cm) and weight 65 kg (50–95 kg). Examination of basic coagulation values under regular standard laboratory conditions at a temperature of 37°C performed as part of the previous study (PT, aPTT, INR, fibrinogen, thrombin time, Antithrombin III) and platelet count showed no pathological deviation in any participant (Wallner et al., 2020).

### Thromboelastometry

**FIGURE 1:**
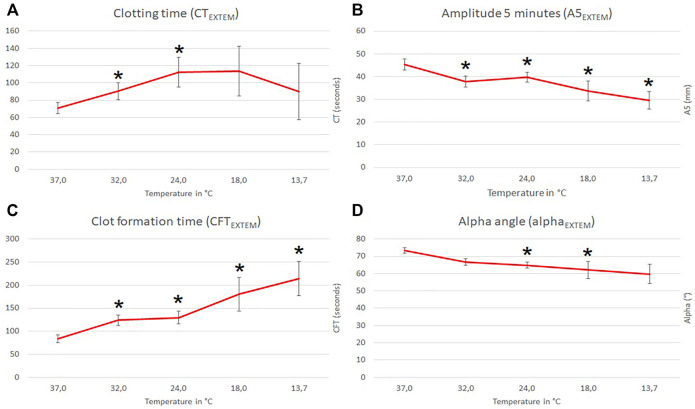
Influence of temperature (x-axis, temperature in degrees Celsius) on ROTEM EXTEM standard parameters (y-axis, seconds in **(A)** and **(C)**; mm in **(B)** and degrees in **(D)**; mean values with respective 95% confidence interval (CI) are shown). * indicates significant differences to baseline at 37°C (*p*-values <0.05).

**FIGURE 2:**
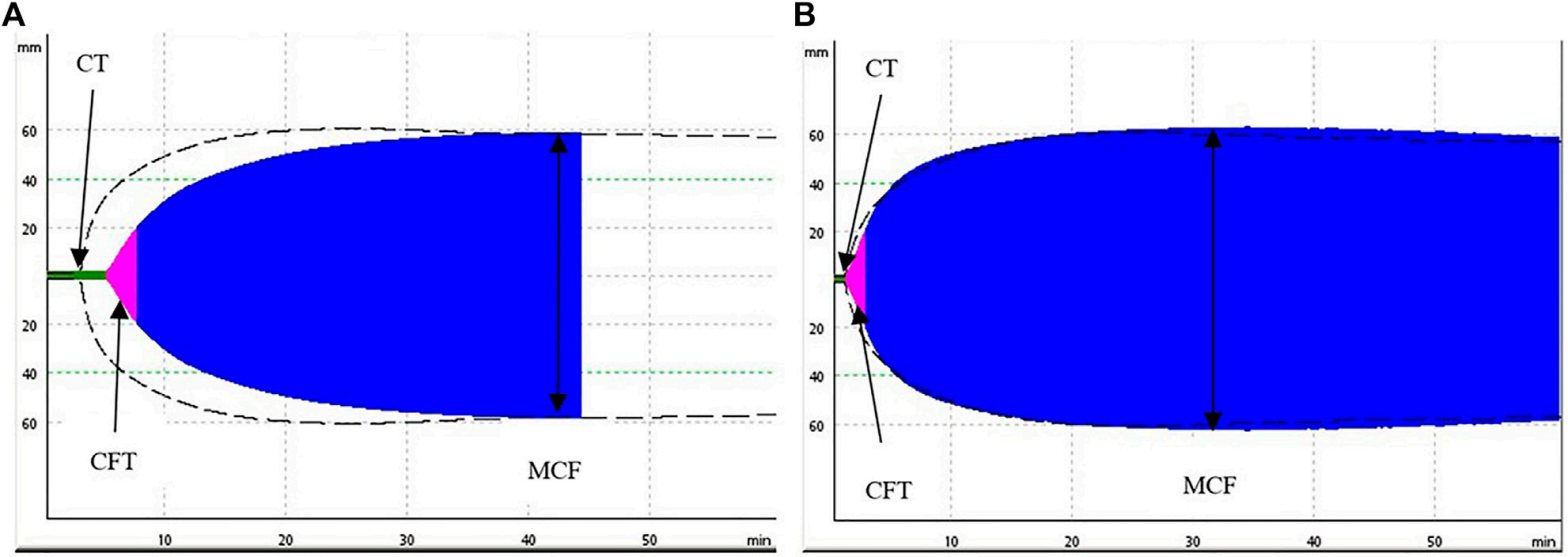
Comparison of two representative ROTEM (ExTEM) tracings measured at 13.7°C **(A)** and at 37°C **(B)**. The **(A)** demonstrates a prolongation of the coagulation time (initiation of the clot) and of the clot formation time (propagation of the clot) when compared to a normal clot formation on the **(B)**, the maximum clot firmness (stiffness of the clot) is unimpaired.

All ROTEM measurements are shown in Supplementary Table S1 and Figure 1. To evaluate the impact on moderate hypothermia we analyzed the effects at 32.0°C in accordance with the Swiss staging (clinical stage 2) (Pasquier et al., 2019). ROTEM standard parameters were defined as EXTEM CT, A5, CFT, MCF and alpha-angle, respectively. Amongst ROTEM standard parameters CT, A5, CFT, and alpha-angle were impaired (ANOVA overall effect, *p* < 0.001) at reduced temperatures below baseline (37°C). CT and CFT at 32.0°C were prolonged to 137 and 159% of the corresponding baseline values (post-hoc, *p* < 0.001). CT was prolonged by 98% at 18°C but reduced to only 34% at 13.7°C; the A5 value continuously dropped to 35% at 13.7°C; the CFT was prolonged 205% at 13.7°C, whereas the alpha angle continuously dropped to 76% at 13.7°C. Compared to baseline, MCF remained unchanged at all tested temperatures (Figure 2).

### Multiple-Electrode Aggregometry

**FIGURE 3:**
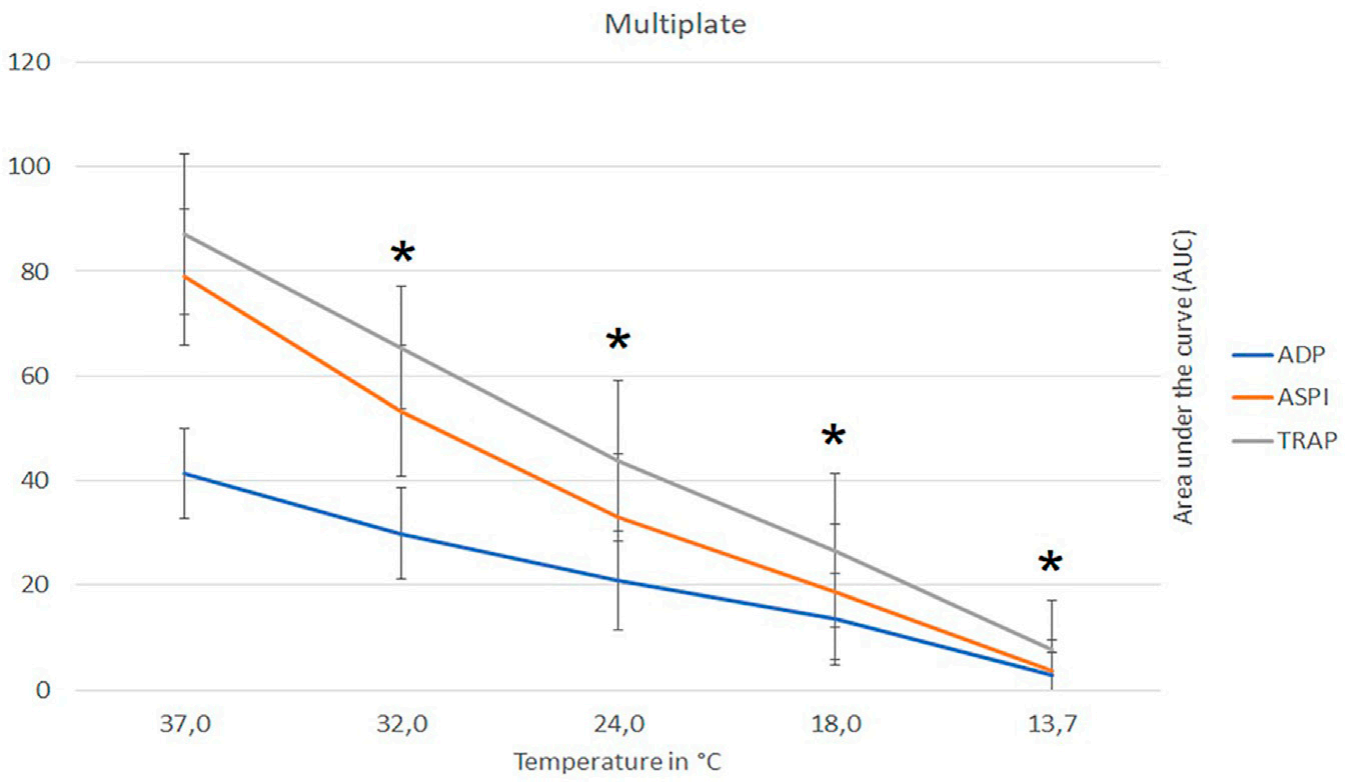
Influence of temperature (x-axis, temperature in degrees Celsius) on platelet function (y-axis, AUC area under the curve) assays ADP, ASPI and Trap, respectively, as measured with multiple-electrode aggregometry (MULTIPLATE). Figure shows mean values with respective 95% confidence interval. AUC, area under the curve; ADP, adenosine diphosphate; ASPI, arachidonic acid; TRAP, thrombin receptor-activating peptide; * indicates significant differences to baseline at 37°C (*p*-values <0.05).

A temperature-dependent reduction in platelet function with decreasing temperature was observed (Supplementary Table S1 and Figure 3). Compared to baseline, the AUC was almost nullified at 13.7°C for all applied tests parameters (ADP, ASPI and TRAP) (ANOVA overall effect, *p* < 0.001, Supplementary Table S1 and Figure 3). At a moderate hypothermia (32.0°C), MULTIPLATE showed significant reduction in thrombocyte activity ADP-AUC to 59% (post-hoc, *p* < 0.001), ASPI-AUC to 66% (post-hoc, *p* < 0.001) and TRAP-AUC to 73% (post-hoc, *p* < 0.001) of the baseline value (37.0°C Figure 3).

## Discussion

This study provides evidence that mild to moderate hypothermia have detrimental effects on coagulation as shown by ROTEM and MULTIPLATE. Severe hypothermia inhibits the function of platelets as shown by the linear decrease in platelet function in MULTIPLATE, but ROTEM does not reflect these changes. Our study shows that viscoelastic tests like ROTEM were unable to reflect the adverse effects of severe hypothermia on platelets, as ROTEM may overestimate clot stability and clot firmness by increased blood viscosity at very low temperatures.

This study is a follow-up study of a previously published analysis, and was designed to systematically analyze clinically important effects of several stages of hypothermia on various components of the coagulation system (Wallner et al., 2020). The preceding study focused on real time live confocal microscopy which enabled to visualize various hypothermia associated changes in the coagulation system as a result of the impairment of thrombin and platelets. In contrast to the previous study, this study specifically focuses on the detection of coagulopathy with point-of-care methods like ROTEM and MULTIPLATE at the different stages of hypothermia. This study was conducted as an *in-vitro* study analyzing whole blood from healthy volunteers. The blood samples were cooled to predefined temperatures and coagulation tests including ROTEM and MULTIPLATE were performed starting from the lowest temperature (13.7°C) to normothermia (37.0°C). ROTEM and MULTIPLATE have been used for the analysis of hypothermia-associated effects on coagulation in previous studies (Kettner et al., 2003; Rundgren and Engström, 2008; Ruzicka et al., 2012). We did not find any significant difference between female and male participants. However, a difference in the coagulation system is described in the literature, with a trend towards higher levels of both fibrinogen and Factor VII in women as well as a higher platelet count and higher platelet reactivity compared to men (Ossei-Gerning et al., 1998; Ranucci et al., 2019).

Patients out of hospital and in the hospital are at risk of hypothermia (Brown et al., 2012; Rauch et al., 2021). Hypothermia may be life threatening in bleeding and traumatized patients. For example, a systematic review on the effects of mild hypothermia on blood loss reported that a drop in core temperature by 1°C is associated with 10–15% increase of intraoperative bleeding in major surgery and 12% increase of packed red blood cells transfusion (Rajagopalan et al., 2008). In a state-wide study performed in Pennsylvania, mortality in multiple trauma patients increased exponentially from <5% at 37°C to almost 50% at <32°C (Wang et al., 2005). Frequently, in trauma patients only standard coagulation test (blood count, PT, aPTT and Fibrinogen-count) are performed.

Those parameters fail to accurately represent the coagulative state of the patient for two reasons. The first reason is that testing of routine parameter is not performed at the patients’ temperature, but samples are warmed by the analyzer by default to 37.0°C. The second reason is that PT or aPTT fail to detect relevant coagulation abnormalities caused by hypothermia. A study showed that ROTEM is better than PT and aPTT in detecting clinically relevant clotting abnormalities and pointed out that there were no significant changes in PT or aPTT at 32.0°C (Martini et al., 2008). Another recent study confirmed that standard coagulation parameters (PT and aPTT) do neither correspond with ROTEM results nor with patients’ blood loss. However, a significant correlation (*p* = 0.001) was seen between fibrinogen levels and alpha angles (Sharma et al., 2018). Thus, we assume that our study may be a valuable contribution to accurately assess hypothermia-associated coagulopathy.

In the present study, the proposed temperature range for mild hypothermia (clinical stage 1 in the Swiss staging system) is <35–32°C (Pasquier et al., 2019). Using the criteria of hypothermia, we observed that all ROTEM parameters (EXTEM CT, A5, CFT, MCF and alpha-angle) were significantly impaired in mild hypothermia below baseline (37°C). A drop in blood temperature to 32°C resulted in a significant reduction of CT, CFT, A5 and the alpha-angle.

An *in vitro* study that compared coagulation at 36°C with coagulation at 32°C using ROTEM confirmed that coagulation was impaired, as expressed by an increased clotting time by 50% at 32°C (Kettner et al., 2003). The same study stated that clot formation was delayed (alpha-angle of 52.9° at 32.0°C vs. 61.8° at 36.0°C) in temperature-adjusted measurements, which indicates that hypothermia reduces plasmatic coagulation and platelet reactivity, but it showed no change if the test temperature was performed at 37°C (Kettner et al., 2003). Mild induced (therapeutic) hypothermia is used for neuroprotection in patients successfully resuscitated after cardiac arrest. In these patients, a body temperature between 36.0 and 33.0 ± 1°C is targeted after the cardiac arrest (Hypothermia after Cardiac Arrest Study Group, 2002; Holzer et al., 2005; Rout et al., 2020; Dankiewicz et al., 2021; Nolan et al., 2021). Furthermore, mild hypothermia is currently undergoing studies in several other indications, e.g., after neonatal asphyxia, myocardial infarction, and traumatic brain injury (Shankaran, 2009; Crossley et al., 2014; Datta, 2017).

The impact of induced hypothermia on coagulation within the range used after cardiac arrest produced minimal but significant prolongation of CT (at 34.0°C) in the study by Rundgren et al. A randomized controlled trial assessing blood loss and transfusion requirements in patients undergoing total hip arthroplasty have shown that intra- and postoperative blood loss was significantly higher in the patients with lower core temperatures: 2.2 versus 1.7 L, (*p* < 0.001) when temperature was decreased from 36.4 to 35.0°C (Schmied et al., 1996; Rundgren and Engström, 2008). However, a study by Wolberg et al. noted that thrombin generation is not affected when temperature is decreased from 37.0 to 33.0°C (Wolberg et al., 2004).

In severe hypothermia (18 and 13.7°C) this study confirmed increasingly impaired ROTEM parameters. The CT was almost doubled compared to baseline values at 18°C. MULTIPLATE results were also significantly impaired and increasing malfunction was evident with decreasing temperature (Supplementary Table S1), resulting in an almost 100% reduction of the AUC at 13.7°C for all applied tests parameters (ADP, ASPI and TRAP). Impairment of coagulation factor enzymatic activity at low temperature is known in the literature, but these studies do not report the degree of impairment *in vivo* (Reed et al., 1990; Reed et al., 1992; Meng et al., 2003). As shown by the present data, hypothermia has detrimental effects on platelet but also on coagulation factors. Hypothermia has a significant negative impact on thrombin generation since it primarily inhibits the initiation phase of thrombin generation (Martini, 2009). The physiological effect of thrombin as a promotor of coagulation factors V, VIII and XI is inhibited. A study performed on survivors after out-of-hospital cardiac arrest treated with targeted temperature management (TTM) at 33 ± 1°C confirmed that TTM impaired thrombin generation. These studies support the findings of our study. Even though we did not measure thrombin directly, we demonstrated the detrimental effects of hypothermia on thrombin generation indirectly through a prolonged CT, a change in alpha-angle and significantly reduced platelet function as demonstrated in MULTIPLATE. A previous study described dynamic changes in the metabolism of fibrinogen during hypothermia. Hypothermia at 32°C primarily inhibits the initiation phase and decreases fibrinogen synthesis as compared to normothermia (Martini, 2007; Martini, 2009; Martini, 2010).

Severe hypothermia, besides having a significant impact on humoral coagulation, is further impairing platelet count and platelet function, adding to the negative consequence on all parts of coagulation. In our study, MULTIPLATE was used to analyze the temperature-associated effect on platelets. We found a significant correlation between low temperatures and platelet dysfunction. At normothermia platelet activity was normal but started to decrease with lower temperature, with almost no detectable platelet activity at and below 18.0°C.

Controversy continues concerning platelet function at mild and moderate hypothermia. A study performed by Scharbert et al. stated that in clinical stage 1 hypothermia (34°C), overall platelet aggregation was increased compared to normothermia (37°C) (Scharbert et al., 2010). However, in severe hypothermia the negative impact on platelet mobility and aggregation is unquestioned (Patt et al., 1988; Van Poucke et al., 2014; Jeppesen et al., 2018). Wolberg et al. stated that mainly platelets and tissue factor-bearing cells can potentially be affected by temperature (Wolberg et al., 2004). Also, platelet aggregation and adhesion were significantly reduced at 33.0°C by comparison with 37.0°C (platelet aggregation size 46.5 at 37.0°C vs. 28.1 at 33.0°C). Below 33°C, however, both enzyme activity and platelet function were reduced (Wolberg et al., 2004). The detrimental effects of hypothermia on platelet function are of utmost importance in multiple trauma patients. Tissue damage and shock-related hypoperfusion in multiple trauma render platelets hyporesponsive (Neal, 2020; Vulliamy et al., 2021). Traumatic brain injury creates a hypocoagulable state by depleting clotting factors systemically and provoking platelet inhibitors (Maegele et al., 2017). The effect of hypothermia and multiple trauma are synergistic.

The present study showed inconsistent effects of hypothermia on platelets by ROTEM and MULTIPLATE. MCF in EXTEM was unchanged even at lowest temperature of 13.7°C but significant reductions were shown in all the MULTIPLATE measurements. MCF represents the strength and firmness of the clot, to which platelets play a substantial role. The unchanged MCF in this study might lead to the false conclusion that the hypothermic patient still has intact clot formation and platelets. The study by Rundgren et al. found that MCF was the variable least impaired at low temperatures (25°C) and concluded that the hypothermia-associated effect is more likely to be related to humoral coagulation factors (Rundgren and Engström, 2008). The authors further argued that this finding suggests that a general impairment of procoagulant enzyme systems occurs that affect hemostatic and platelet activity.

Another study by Kettner et al. found no change in clot strength, which is the variable equivalent to MCF in ROTEM (Kettner et al., 2003). Also, Solomon et al. pointed out that MCF was inappropriate as a parameter because clot amplitude and clot elasticity were not properly correlated (Solomon et al., 2015). Their study indicated that platelet contribution to clot strength was underestimated by using MCF and that MCF also remains unchanged across a range of platelet counts (from 10,000 to 100,000/μL). This was in accordance with previous studies and can be explained by the fact that ROTEM measures the viscosity of the blood and the clot. Cooled blood demonstrates a higher viscosity and ROTEM, as a viscometer, measures false normal values especially in MCF (Wallner et al., 2020).

ROTEM is more frequently used as point-of-care testing and has already been integrated into treatment algorithms for massive hemorrhage. Considering only CT and MCF as clinically important parameters, clinically relevant hypothermia induced impairment of platelets will be missed.

## Limitations

In 2020 a case report of a 2-year old boy surviving neurologically intact an unintentional drop to a core temperature of 11.8°C was reported (Mroczek et al., 2020). However, the clinical relevance between the 13.7°C as lowest temperature used in our study and the 11.8°C is minor because only two individuals unintentionally reached and survived these temperatures. We are not aware of any differences in the function of coagulation at these ultra-deep temperatures and only very few patients will ever reach these temperatures. In order to cool the (normothermic) blood samples to the set temperature we had to incubate all the samples of our healthy volunteers for 30 min in a water-bath at the desired temperature before we commenced the analysis. We acknowledge the fact that the situation in hypothermic trauma patients is different because the blood samples in hypothermic patients already represent the exact temperature of the patient and do not need to be cooled further. The blood samples were systematically cooled to specifically predefined temperatures and coagulation tests were performed starting from 13.7 to 37.0°C. By starting from the lowest temperature, we wanted to demonstrate that the adverse effect on coagulation are first and foremost caused by the low temperature and not by other impairing factors, such as storing of the blood samples. Using a single blood sample at every temperature stage would have been better. We further acknowledge that in multiple trauma patients several factors impair the coagulation system such as hemorrhage, inflammation, acidosis, but also therapeutic interventions like volume resuscitation and the administration of drugs which act directly or indirectly on the coagulation system. We acknowledge that low temperatures *in-vitro* do not directly resemble hypothermia *in-vivo*. The clinical picture of hypothermia constitutes of numerous pathophysiological changes like inflammation, release of acute phase proteins and cytokines, and activation of the complement system. In the present study we aimed to isolate the distinct effects of low temperatures on platelets and the hemostatic system. The difference between MCF in ExTEM and FibTEM to subtract the fibrinogen component and thereby estimate the contribution of platelets to the clot has not been calculated as a surrogate marker.

## Conclusion

Hypothermia impairs coagulation by prolonging coagulation clotting time and by decreasing the velocity of clot formation in ROTEM measurements. MULTIPLATE testing confirms a linear decrease in platelet function with decreasing temperatures, but ROTEM fails to adequately detect hypothermia induced impairment of platelets.

## Data Availability

The raw data supporting the conclusion of this article will be made available by the authors, without undue reservation.
