# Supplementary material for: Hypothermia Induced Impairment of Platelets: Assessment With Multiplate vs. ROTEM—An In Vitro Study
Source: Front Physiol. 2022 Mar 29;13:852182. doi: 10.3389/fphys.2022.852182 (PMC9002345; doi:10.3389/fphys.2022.852182)
Supplement: Supplementary file 1 [file Table1.docx]

|  | **Temperature** | |  |  | **37°C** | | |  | | | | **32°C** | | |  | | | **24°C** | | |  | | | **18°C** | | |  | | | **13.7°C** | | |
| --- | --- | --- | --- | --- | --- | --- | --- | --- | --- | --- | --- | --- | --- | --- | --- | --- | --- | --- | --- | --- | --- | --- | --- | --- | --- | --- | --- | --- | --- | --- | --- | --- |
|  | **Parameter** | |  | **Mean** | |  | **SD** | |  | | **Mean** | |  | **SD** | |  | **Mean** | |  | **SD** | |  | **Mean** | |  | **SD** | |  | **Mean** | |  | **SD** |
| **ROTEM** |  |  |  |  | |  |  | |  | |  | |  |  | |  |  | |  |  | |  |  | |  |  | |  |  | |  |  |
| **EXTEM** | **CT** | **sec** |  | 71 | |  | 14 | |  | 90 | | |  | 21 | |  | 112 | |  | 36 | |  | 113 | |  | 61 | |  | 90 | |  | 69 |
|  | **A5** | **mm** |  | 45 | |  | 5 | |  | 38 | | |  | 5 | |  | 40 | |  | 5 | |  | 34 | |  | 9 | |  | 30 | |  | 8 |
|  | **CFT** | **sec** |  | 83 | |  | 18 | |  | 124 | | |  | 25 | |  | 130 | |  | 29 | |  | 180 | |  | 77 | |  | 214 | |  | 78 |
|  | **MCF** | **mm** |  | 62 | |  | 4 | |  | 58 | | |  | 5 | |  | 60 | |  | 4 | |  | 60 | |  | 4 | |  | 60 | |  | 4 |
|  | **alpha** | **°** |  | 3 | |  | 74 | |  | 4 | | |  | 66 | |  | 4 | |  | 65 | |  | 11 | |  | 63 | |  | 12 | |  | 56 |
|  |  |  |  |  | |  |  | |  |  | | |  |  | |  |  | |  |  | |  |  | |  |  | |  |  | |  |  |
| **FIBTEM** | **CT** | **sec** |  | 70 | |  | 11 | |  | 94 | | |  | 20 | |  | 104 | |  | 44 | |  | 99 | |  | 68 | |  | 67 | |  | 49 |
|  | **A5** | **mm** |  | 11 | |  | 3 | |  | 11 | | |  | 3 | |  | 11 | |  | 3 | |  | 10 | |  | 3 | |  | 10 | |  | 3 |
|  | **MCF** | **mm** |  | 13 | |  | 4 | |  | 13 | | |  | 3 | |  | 12 | |  | 3 | |  | 11 | |  | 3 | |  | 12 | |  | 3 |
|  | **alpha** | **°** |  | 67 | |  | 8 | |  | 64 | | |  | 7 | |  | 66 | |  | 9 | |  | 51 | |  | 19 | |  | 45 | |  | 21 |
|  |  |  |  |  | |  |  | |  |  | | |  |  | |  |  | |  |  | |  |  | |  |  | |  |  | |  |  |
| **INTEM** | **CT** | **sec** |  | 201 | |  | 40 | |  | 260 | | |  | 48 | |  | 347 | |  | 281 | |  | 305 | |  | 78 | |  | 301 | |  | 73 |
|  | **A5** | **mm** |  | 42 | |  | 4 | |  | 34 | | |  | 6 | |  | 41 | |  | 4 | |  | 39 | |  | 4 | |  | 38 | |  | 4 |
|  | **CFT** | **sec** |  | 161 | |  | 311 | |  | 143 | | |  | 61 | |  | 196 | |  | 307 | |  | 111 | |  | 22 | |  | 113 | |  | 17 |
|  | **MCF** | **mm** |  | 56 | |  | 8 | |  | 54 | | |  | 6 | |  | 57 | |  | 4 | |  | 56 | |  | 4 | |  | 56 | |  | 5 |
|  | **alpha** | **°** |  | 73 | |  | 3 | |  | 67 | | |  | 4 | |  | 71 | |  | 2 | |  | 69 | |  | 3 | |  | 68 | |  | 3 |
|  |  |  |  |  | |  |  | |  |  | | |  |  | |  |  | |  |  | |  |  | |  |  | |  |  | |  |  |
| **Multiplate** |  |  |  |  | |  |  | |  |  | | |  |  | |  |  | |  |  | |  |  | |  |  | |  |  | |  |  |
| **ADP** | **AUC** | **U** |  | 41 | |  | 18 | |  | 30 | | |  | 19 | |  | 21 | |  | 20 | |  | 14 | |  | 19 | |  | 3 | |  | 10 |
| **ASPI** | **AUC** | **U** |  | 79 | |  | 28 | |  | 53 | | |  | 27 | |  | 33 | |  | 26 | |  | 19 | |  | 28 | |  | 4 | |  | 13 |
| **TRAP** | **AUC** | **U** |  | 87 | |  | 33 | |  | 65 | | |  | 25 | |  | 44 | |  | 33 | |  | 27 | |  | 32 | |  | 8 | |  | 21 |

***Table 1.*** Mean and std. deviation (SD) of the results for standard parameters of thromboelastometry and multiple-electrode aggregometry for the various temperatures. A5, clot amplitude 5 min after CT; ADP, adenosine diphosphate; alpha, alpha angle; ASPI, arachidonic acid; AUC, area under the curve; CFT, clot formation time; CT, coagulation time; MCF, maximum clot formation; TRAP, thrombin receptor-activating peptide.
